# The potential role of robust face representations learned within families when searching for one’s child in a crowd

**DOI:** 10.1038/s41598-024-66964-4

**Published:** 2024-07-22

**Authors:** Yousuke Kawachi, Aiko Murata, Miho S. Kitamura, Ryoko Mugitani

**Affiliations:** 1 Department of Psychology, Graduate School of Arts and Letters, Tohoku University, 27-1, Kawauchi, Aoba-ku, Sendai, 9808576 Japan; 2 NTT Communication Science Laboratories, NTT Corporation, Atsugi, Japan; 3 IdeaLab Inc., Tokyo, Japan; 4 Waseda Institute for Advanced Studies, Waseda University, Tokyo, Japan; 5 Department of Psychology, Faculty of Integrated Arts and Social Sciences, Japan Women’s University, Tokyo, Japan

**Keywords:** Own child’s face, Family member’s faces, Visual search, Visual attention, Overlearning, Psychology, Human behaviour

## Abstract

Own child’s face is one of the most socially salient stimuli for parents, and a faster search for it than for other children’s faces may help provide warmer and more sensitive care. However, it has not been experimentally examined whether parents find their child’s face faster. In addition, although own child’s face is specially processed, the search time for own child’s face may be similar to that for other socially salient stimuli, such as own or spouse’s faces. This study tested these possibilities using a visual search paradigm. Participants (parents) searched for their child’s, own, spouse’s, other child’s, same-sex adult’s, or opposite-sex adult’s faces as search targets. Our findings indicate that both mothers and fathers identified their child’s face more quickly than other children’s faces. Similarly, parents found their own and spouse’s faces more quickly than other adults’ faces. Moreover, the search time for family members’ faces increased with the number of faces on the search display, suggesting an attentional serial search. These results suggest that robust face representations learned within families and close relationships can support reduced search times for family members’ faces.

## Introduction

Searching for and finding faces is essential for smoothing our social interactions. A child’s face is considered a unique stimulus that induces feelings of care and empathy in adults. As parents must nurture and protect their children, they should be tuned and sensitive to signals from their children to protect them from dangerous situations and to provide appropriate help. Non-family infant faces seem to be salient social stimuli that elicit feelings of care and an empathic approach in them^1–3^ and capture adults’ attention^4^. Thus, own children’s faces can be the most important targets that parents should find rapidly and accurately and devote their limited attentional resources.

Previous neuroimaging studies support the idea that own child’s face is special. Studies involving mothers have reported that brain areas (midbrain, ventrolateral prefrontal cortex, insula, amygdala, and striatum) show higher activation when looking at the face of their own child (for a review, Rigo et al.^5^). These brain areas are associated with the detection of salience in own child, maternal motivation/reward and approach/avoidance behavior modulation, and social emotion regulation^6^. Human parenting induces adjustments in behavioral responses to own children’s faces. However, to the best of our knowledge, few behavioral studies have verified whether parents acquire specific perceptual sensitivities toward their child’s face and detect them more quickly and precisely.

A study by Tong and Nakayama^7^ on visual search reported a facilitation effect for participants’ own faces in a visual search task in which they searched for their own faces among others’ as distractors. They suggested that one’s own face is highly overlearned by various stimulus conditions and contexts to form a robust representation that facilitates its visual processing. They assumed that robust representations lead to rapid asymptotic processing, meaning that the reduction of search response time (RT) is negligible through trials. They theoretically expected that close family members’ or friends’ faces should be highly overlearned, leading to facilitation effects in similar visual search tasks in which parents search and detect highly overlearned faces, such as their own, spouses, and children’s faces, more quickly than that of others.

As Tong and Nakayama^7^ described, searching for one's child's face may be quicker than that for other children's faces. Moreover, although neuroimaging studies focus on the specialized processing of own children's faces, previous behavioral studies suggest that the search times for one’s own and spouse’s faces, which are also socially salient stimuli, may be comparable to those of one’s children’s faces^7,8^. Thus, it is unclear whether behavioral responses to one’s child’s face are quicker than those to own or spouse’s face^9^. Therefore, following Tong and Nakayama^7^, we hypothesized that parents search for and detect highly overlearned faces, such as their child’s, spouse’s, and own faces, more quickly than that of others. In addition, the RT would slightly reduce through trials for highly overlearned faces.

Although previous studies have assumed that gazing, detecting, and searching for infants’ faces is mediated by attention (for a review, Lucion et al.^4^), do parents attentively search for their children? Treisman and Gelade^8^ state that “Searching for a face, even as familiar as one’s own child, in a school photograph, can be a painstakingly serial process and focused attention is certainly recommended in proof reading and instrument monitoring.” However, studies on conventional visual searches for the faces of own children and other family members are lacking. Therefore, we had set sizes consisting of two, four, or six children faces to examine the set-size effect; that is, whether the RT to find the target increases as the number of distractors increases. Previous studies on visual search tasks have attributed the set-size effect to limited covert attentional processes^10^. When performance is not affected by the number of distractors, it is a pre-attentive parallel search, but when it deteriorates as the set size increases, it is a covert attentional serial search^8,11,12^.

Processing one’s own child’s face yields another question: are there any differences between maternal and paternal information processing of own child’s face? Previous studies have focused on parental sensitivity, which involves detecting signals emitted by children, understanding them correctly, and responding appropriately to them^13^. Experimenters in previous studies have recorded semi-structured free-play interactions between children and their mothers and between children and their fathers. Coders rated their behaviors in videos according to parental sensitivity criteria to assess the sensitivity of mothers and fathers^14–16^. Although there is no agreement on the differences in parental sensitivity between mothers and fathers, previous studies have indicated that mothers are generally more sensitive toward their children than fathers (e.g., Hallers-Haalboom et al.^17^). Thus, the mother/father factor is undoubtedly important.

The present study used the visual search paradigm which directly elucidates whether parents quickly detect their child’s faces among other distractors to examine the specificity and possible maternal/paternal differences in visual processing of own child’s face. Given that parental traits such as caregiving and mother–child bonding are related to infants' face processing^18,19^, we measured childcare involvement and parental attitudes using questionnaires to explore the relationship with visual search performance.

## Methods

### Ethical statements

This study was approved by the Ethical Committee of NTT Communication Science Laboratories (H27-020) and conducted according to the principles of the Declaration of Helsinki. Written informed consent was obtained from all participants for their participation and use of their and their child’s pictures.

### Participants

Eighteen Japanese mother-father pairs raising 2–3 year-old children participated in this study (mean age: 36.53, SD: 4.75). The participants were compensated monetarily for their contribution in the study. All participants had either normal or corrected-to-normal vision and scored below 52.16, an upper outlier (average + 2SD) as indicated by Kanayama et al.^20^, on the Japanese version of the Congenital/Hereditary Prosopagnosia Screening Scale.

We conducted a statistical power analysis to verify whether the current sample size was adequate to detect the main effect of own-face facilitation^7^ or the interaction between the presence/absence of daily learning about children’s faces and target face age^21^ in visual search using the Bias and Uncertainty Corrected Sample Size (BUCSS) package in R^22^. This package uses the reported *F* value and sample size from a previous study to generate the sample size necessary to obtain the desired test power and alpha levels. We used *F*(1, 7) = 6.7 from Experiment 1 in the study by Tong and Nakayama^7^, and *F*(1, 32) = 7.006 from Experiment 2 conducted by Macchi Cassia et al.^21^, with an alpha (0.05), a level of assurance (0.8), and a desired power (0.8). The analysis generated sample sizes of six and 10. Therefore, a sample size of 18 participants per group should provide a sufficient statistical power.

### Apparatus

All stimuli were generated on a PC (VAIO Z; VAIO Cooperation: Nagano, Japan) in MATLAB 2015a (The MathWorks, Inc., Natick, MA, USA) using the Cogent Graphics package (http://www.vislab.ucl.ac.uk/cogent.php). All stimuli were displayed on a CRT monitor (GDM-F520; Sony, Tokyo, Japan) at a refresh rate of 60 Hz and resolution of 1024 × 768 pixels. The monitor had gamma-corrected luminance.

### Stimuli and procedures

Target and unfamiliar distractor face stimuli were grayscale photographs taken using a digital video camera (HC-WX970M; Panasonic, Tokyo, Japan). Photographs of the fathers, mothers, and children were taken on the day of the experiment. Photographs of unfamiliar adults and age-matched children were captured before the experiment. A face was detected from a photograph using the MATLAB built-in Viola-Jones algorithm to control the spatial configuration across face stimuli, and was trimmed with an oval window (height: 1.60° of visual angle; width: 1.37° of visual angle). The average luminance (60.40 cd/m^2^) across face stimuli was adjusted using the SHINE toolbox^23^ (Willenbockel et al.^23^). The hair was removed using Adobe Photoshop. All the faces were photographed and adjusted under identical conditions to minimize recognition based on noticeable figural differences between faces. We captured several pictures of the participants and carefully selected the one with the most neutral expression.

Each participant sat approximately 57 cm away from the display and used a chin rest to stabilize their visual field. Each participant searched for a target of their own face, their spouse's face, their own child's face, a same-sex adult's face (identical for all participants), an opposite-sex adult's face (identical for all participants), or another person's child's face (identical for all participants) among distractors in separate trial blocks. The distractors were randomly selected for each trial from the set of six same-sex adults' faces, six different-sex adults' faces, or six other people's children's faces, depending on the target-type condition, and the same face was never presented more than once on a given trial. The number of distractor faces was 18 in total. The number of times each face image was presented was not equal. The six combinations of targets and distractors consisted of two ownership conditions (own and other) and three target-type conditions (same-sex adult, opposite-sex adult, and child) as follows: (1) own-face target and same-sex adult-face distractors, (2) spouse's face target and opposite-sex adult-face distractors, (3) own child's face target and another's child-face distractors, (4) same-sex adult-face target and same-sex adult-face distractors, (5) opposite-sex adult-face target and opposite-sex adult-face distractors, and (6) another person's child's face target and another person's child-face distractors. The child-face distractor set comprised equal numbers of same-sex and opposite-sex children's faces. Although the sex of the randomly selected child-face distractors was not matched for each trial and set size, post-experiment informal interviews with participants indicated that they could not identify the sex based on the children's faces.

**Figure 1:**
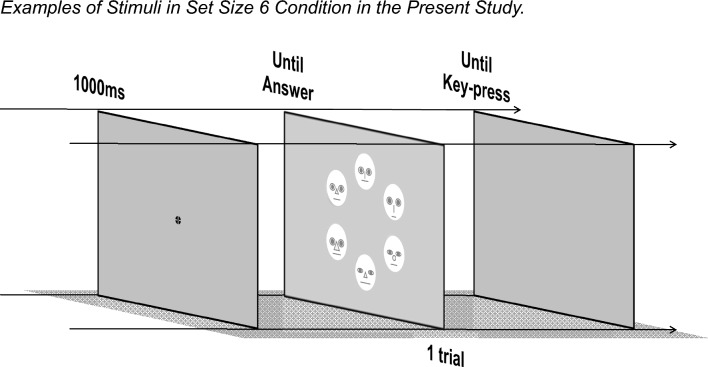
Examples of stimuli in set size 6 condition in the present study. *Note.* The actual facial stimuli were photographs and not line drawings. Details of the facial stimuli are described in the "Methods" section.

For each trial (Fig. 1), a black fixation point appeared at the center of the display against a gray background. After 1000 ms, 2, 4, or 6 face stimuli (set sizes 2, 4, or 6) were located at 0° and 180°, 45°, 135°, 225° and 315°, or 30°, 90°, 150°, 210°, 270°, and 330° of a virtual circle with a radius of 5.05° of visual angle. Each facial stimulus was randomly assigned to one of several possible locations. All three set sizes occurred at equal frequency and were randomly ordered within a given trial block. These face stimuli appeared until participants pressed one of the two possible keys on the keyboard to indicate whether the target was present or absent as quickly and accurately as possible.

The target face was shown at the beginning of each trial block^7^ for 30 s. This duration was determined based on a preliminary experiment, ensuring that participants could perform the task without undue task difficulty. Each trial block included 120 randomly presented trials: 60 target-present and 60 target-absent trials. The 60 trials consisted of 3 set-size conditions (set-sizes 2, 4, and 6) × 20 repetitions. The combination of target and distractor presented in each trial block was one of six types (2 ownership conditions [own and other] × 3 target-type conditions [same-sex adult, opposite-sex adult, and child]). Each trial block was performed in duplicate. The order of the 12 trial blocks was counterbalanced across participants.

### Questionnaires

Each participant completed questionnaires regarding childcare participation as parents. The questionnaires included weekday child-rearing hours, off-day child-rearing hours, and 11 items on childcare involvement (i.e., talking, playing, going out together, staying at home together, reading picture books, taking care of meals, toileting, bathing, sleeping, dressing, and hospital attendance). The 11 items were rated on a four-point scale from never (0) to all the time (3). High overall scores indicated high involvement in daily child rearing.

Additionally, the Maternal Attitude Scale^24^ was administered. This scale measures positive and negative attitudes toward being a mother. The original version had 12 items, six for positive attitude (e.g., “I like being a mother”) and six for negative attitude (e.g., “Because I am a mother, my actions are quite restricted”). We omitted one item each for positive and negative attitudes, owing to the lowest factor load^25^. In addition, the wording was altered from “mother” to “parent” to ensure applicability to both mothers and fathers in the present study. The items were rated on a four-point scale from disagree (1) to agree (4).

The Feelings towards the Baby Scale^26^ was used to measure both approach and avoidance feelings toward infants. It consists of 28 items: 14 items are composed of adjectives indicating approach feelings of proximity and acceptance of infants (e.g., sweet, pretty) and 14 avoidance items are composed of adjectives indicating avoidance feelings of denial and rejection of infants (e.g., scary, burdensome). The items were rated on a four-point scale from not so (0) to exactly so (3).

### Data analysis

We calculated the correct RT from the appearance of the face stimuli until the participant pressed a key and the response accuracy. We conducted a five-way mixed repeated-measures analysis of variance (ANOVA) for the correct RT and response accuracy with the sex of parent (mother or father) as a between-subjects factor and the ownership (own or other), target type (child, same-sex adult or opposite-sex adult), set size (2, 4, or 6), and target presence (presence or absence) as within-subjects factors.

We conducted a time-course analysis to investigate the possible overlearning of self-related faces. We divided 20 target-present trials for each set-size condition in each trial block into five trial bins, consisting of four target-present trials. We then calculated the mean correct RT for each trial bin. We analyzed the RTs scaled as ratios of bin1:bin1, 2, 3, 4, and 5 RTs with a five-way mixed repeated-measures ANOVA with the sex of parent as a between-subjects factor, and the ownership, target type, and set size as within-subjects factors to examine the magnitude of the learning effect across trials.

We conducted exploratory correlational analyses considering the questionnaire scores, which may be related to search performance. To create an index summarizing search performance, we calculated each participant’s slopes and intercepts of search functions in the own- and other-child conditions using linear regression and estimated the set size 1 RT^7^.

## Results

### General analysis

**Figure 2:**
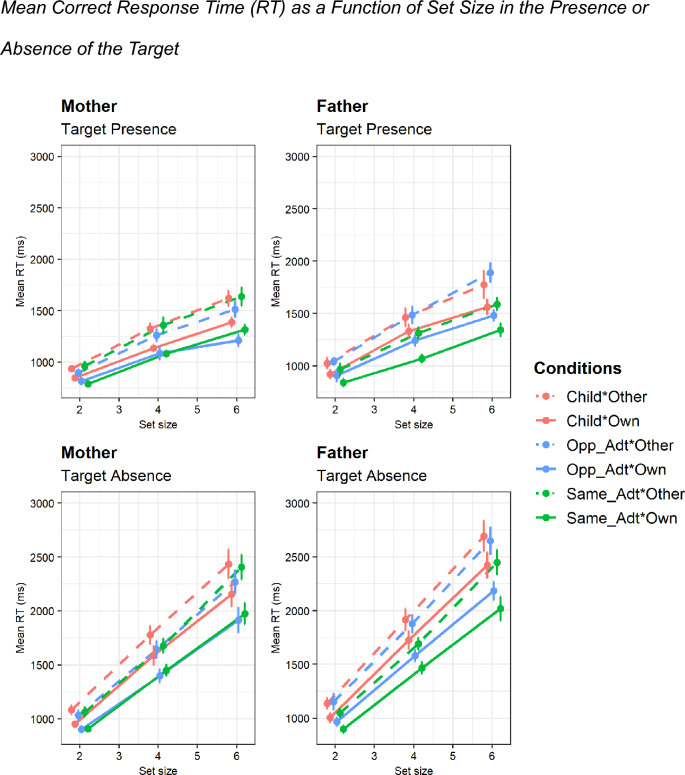
Mean correct response time (RT) as a function of set size in the presence or absence of the target. *Note.* Error bars denote the standard error of the mean (SEM). Opp_Adt, Opposite-sex adult, Same_Adt, Same-sex adult.

Table 1 shows the results of ANOVA for correct RT. Based on our hypothesis, we focused on the main effects of mother/father, own/other, and child/same-sex adult/opposite-sex adult and the interaction among these factors (Fig. 2). Moreover, if visual search is attentional, a set-size-related interaction would be obtained. For clarity, only significant results were reported here. The analysis revealed the significant main effects of ownership, *F*(1, 34) = 90.67, *p* < 0.001, *η*_*p*_^2^ = 0.73, target type, *F*(2, 68) = 9.632, *p* < 0.001, *η*_*p*_^2^ = 0.22, and set size, *F*(1.10, 37.51) = 770.04, *p* < 0.001, *η*_*p*_^2^ = 0.96. Although the interaction between ownership and target type was not significant, the set size related two-way interactions were significant with target type, *F*(3.18, 107.99) = 6.61, *p* < 0.001, *η*_*p*_^2^ = 0.16, and with ownership, *F*(1.38, 47.02) = 58.17, *p* < 0.001, *η*_*p*_^2^ = 0.63. The sex of parent-related three-way interaction of the sex of parent, target type, and set size was also significant, *F*(3.18, 107.99) = 4.96, *p* < 0.005,*η*_*p*_^2^ = 0.13. Post-hoc analyses with Bonferroni correction indicated that self-related faces were found quicker than other faces across all set-size conditions (set size 2: *p* < 0.001, Cohen’s *d* = 0.65; set size 4: *p* < 0.001, Cohen’s *d* = 0.59; set size 6: *p* < 0.001, Cohen’s *d* = 0.56). In addition, the search RT increased with the set size under both the own and other conditions (own × set size 4 relative to set size 2: *p* < 0.001, Cohen’s *d* = 1.66; own × set size 6 relative to set size 4: *p* < 0.001, Cohen’s *d* = 1.87; own × set size 6 relative to set-size 2: *p* < 0.001, Cohen’s *d* = 0.98; other × set size 4 relative to set size 2: *p* < 0.001, Cohen’s *d* = 1.66; other × set size 6 relative to set size 4: *p* < 0.001, Cohen’s *d* = 1.97; other × set-size 6 relative to set-size 2: *p* < 0.001, Cohen’s *d* = 1.07). Regarding the three-way interaction, regardless of the ownership conditions, fathers had significantly shorter RTs for the same-sex search than for the child search for each set size (set size 2: *p* < 0.005, Cohen’s *d* = 0.38; set size 4: *p* < 0.001, Cohen’s *d* = 0.63; set size 6: *p* < 0.001, Cohen’s *d* = 0.41) or opposite-sex adult search for each set size (set size 2: *p* < 0.05, Cohen’s *d* = 0.33; set size 4: *p* < 0.005, Cohen’s *d* = 0.45; set size 6: *p* < 0.005, Cohen’s *d* = 0.34), but mothers did not differ in RTs for the child and same-sex search (although RTs in the search for the opposite-sex adult were significantly shorter than those for the child in set size 6, *p* < 0.05, Cohen’s *d* = 0.29). In addition, the effect of set size was significant for both fathers and mothers for all targets (all *p*s < 0.001, Cohen’s *d* ranged from 0.79 to 2.26). We did not describe the set size effect in further detail because the general trend of longer RTs with increasing set sizes was similar to the set size effect described earlier for the interaction between set size and ownership conditions. Although outside the scope of this study, mothers had significantly shorter RTs for opposite-sex searches than fathers had in set size 4, *p* < 0.05, Cohen’s *d* = 0.54, and set size 6, *p* < 0.05, Cohen’s *d* = 0.56.

**Table 1:** Analysis of variance (ANOVA) of mean response time (RT).

Source	Mean RT			
	*F* value	*df*	*p* value	Partial eta-square
Sex of parent (SP)	2.10	1, 34	0.16	0.06
Target presence (TP)	**338.23**	**1, 34**	**< 0.001**	**0.90**
TP × SP	0.41	1, 34	0.84	0.001
Target type (TT)	**9.63**	**2, 68**	**< 0.001**	**0.22**
TT × SP	**6.95**	**2, 68**	**< 0.005**	**0.17**
Ownership (OS)	**90.67**	**1, 34**	**< 0.001**	**0.73**
OS × SP	0.06	1, 34	0.80	0.002
Set size (SS)	**770.04**	**1.10, 37.51**	**< 0.001**	**0.96**
SS × SP	3.30	1.10, 37.51	0.07	0.09
TP × TT	**6.61**	**2, 68**	**< 0.005**	**0.16**
TP × TT × SP	0.14	2, 68	0.87	0.004
TP × OS	**6.49**	**1, 34**	**< 0.05**	**0.16**
TP × OS × SP	0.40	1, 34	0.53	0.01
TT × OS	2.39	2, 68	0.10	0.07
TT × OS × SP	1.01	2, 68	0.37	0.03
TP × TT × OS	0.08	2, 68	0.93	0.002
TP × TT × OS × SP	0.15	2, 68	0.86	0.004
TP × SS	**58.17**	**1.22, 41.33**	**< 0.001**	**0.88**
TP × SS × SP	0.95	1.22, 41.33	0.35	0.03
TT × SS	**6.61**	**3.18, 107.99**	**< 0.001**	**0.16**
TT × SS × SP	**4.96**	**3.18, 107.99**	**< 0.005**	**0.13**
TP × TT × SS	1.54	2.23, 75.77	0.22	0.04
TP × TT × SS × SP	0.89	2.23, 75.77	0.43	0.03
OS × SS	**58.17**	**1.38, 47.02**	**< 0.001**	**0.63**
OS × SS × SP	0.08	1.38, 47.02	0.86	0.002
TP × OS × SS	2.62	2, 68	0.08	0.07
TP × OS × SS × SP	0.06	2, 68	0.95	0.002
TT × OS × SS	1.61	2.53, 86.09	0.20	0.05
TT × OS × SS × SP	0.27	2.53, 86.09	0.81	0.01
TP × TT × OS × SS	1.95	3.11, 105.71	0.12	0.05
TP × TT × OS × SS × SP	0.16	3.11, 105.71	0.93	0.01

Significant values are in bold.

**Figure 3:**
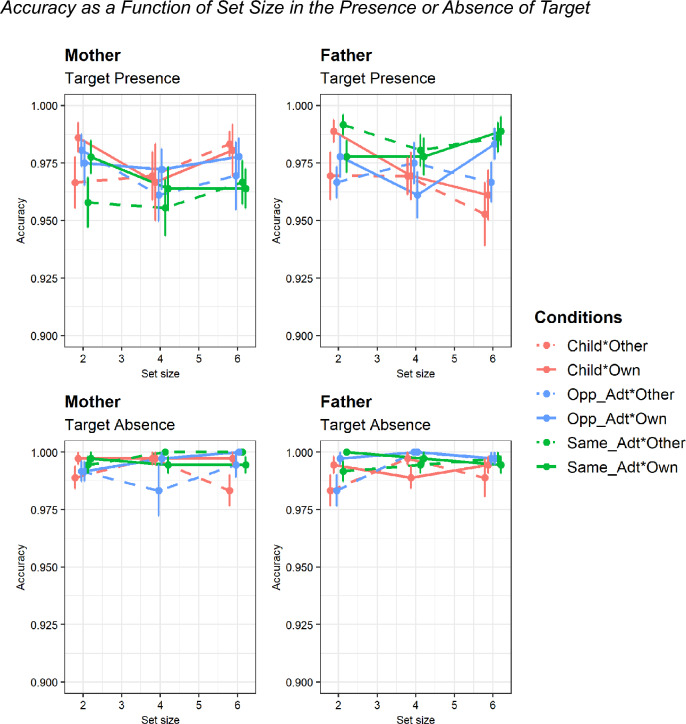
Accuracy as a function of set size in the presence or absence of target. *Note.* Error bars denote the standard error of the mean (SEM). Opp_Adt, Opposite-sex adult, Same_Adt, Same-sex adult.

Regarding accuracy (Fig. 3 and Table 2), the analysis revealed significant main effects of ownership, *F*(1, 34) = 4.32, *p* < 0.05,*η*_*p*_^2^ = 0.11, with slightly higher accuracy in the own condition. The two-way interaction of the sex of parent and target type and three-way interaction of the sex of parent, target type, and target presence were significant, *F*(2, 68) = 4.90, *p* < 0.05, *η*_*p*_^2^ = 0.13 and *F*(2, 68) = 4.61, *p* < 0.05, *η*_*p*_^2^ = 0.12, respectively. Post-hoc analyses with Bonferroni correction indicated that fathers more accurately found target faces of the same sex as than did mothers in the target-present trials,* p* < 0.005, Cohen’s *d* = 0.54. Additionally, fathers were significantly more accurate at finding same-sex adult faces than target children’s faces in the target-present trials, *p* < 0.05, Cohen’s *d* = 0.41. Additionally, fathers and mothers were generally more accurate in the target-present trials than in the target-absent trials (*p-*values ranged from 0.013 to < 0.001, Cohen’s d ranged from 0.51 to 1.04).

**Table 2:** Analysis of variance (ANOVA) of accuracy.

Source	Accuracy			
	*F* value	*df*	*p* value	Partial eta-square
Sex of parent (SP)	0.36	1, 34	0.55	0.10
Target presence (TP)	**65.37**	**1, 34**	**< 0.001**	**0.66**
TP × SP	0.57	1, 34	0.46	0.02
Target type (TT)	0.91	2, 68	0.40	0.03
TT × SP	**4.90**	**2, 68**	**< 0.05**	**0.13**
Ownership (OS)	**4.32**	**1, 34**	**< 0.05**	**0.11**
OS × SP	0.16	1, 34	0.69	0.01
Set size (SS)	0.77	2, 68	0.47	0.02
SS × SP	0.62	2, 68	0.54	0.02
TP × TT	0.10	2, 68	0.90	0.003
TP × TT × SP	**4.61**	**2, 68**	**< 0.05**	**0.12**
TP × OS	0.07	1, 34	0.80	0.002
TP × OS × SP	0.11	1, 34	0.74	0.003
TT × OS	0.91	2, 68	0.40	0.03
TT × OS × SP	0.12	2, 68	0.88	0.003
TP × TT × OS	0.54	2, 68	0.95	0.002
TP × TT × OS × SP	1.06	2, 68	0.35	0.30
TP × SS	**3.98**	**2, 68**	**< 0.05**	**0.11**
TP × SS × SP	0.52	2, 68	0.60	0.02
TT × SS	0.80	4, 136	0.53	0.02
TT × SS × SP	1.10	4, 136	0.36	0.03
TP × TT × SS	0.22	4, 136	0.92	0.01
TP × TT × SS × SP	2.03	4, 136	0.09	0.06
OS × SS	1.92	2, 68	0.15	0.05
OS × SS × SP	0.99	2, 68	0.38	0.03
TP × OS × SS	0.05	2, 68	0.95	0.002
TP × OS × SS × SP	1.22	2, 68	0.30	0.04
TT × OS × SS	0.94	4, 136	0.43	0.03
TT × OS × SS × SP	1.53	4, 136	0.20	0.04
TP × TT × OS × SS	0.81	4, 136	0.52	0.02
TP × TT × OS × SS × SP	0.26	4, 136	0.91	0.01

Significant values are in bold.

### Time-course analysis

**Figure 4:**
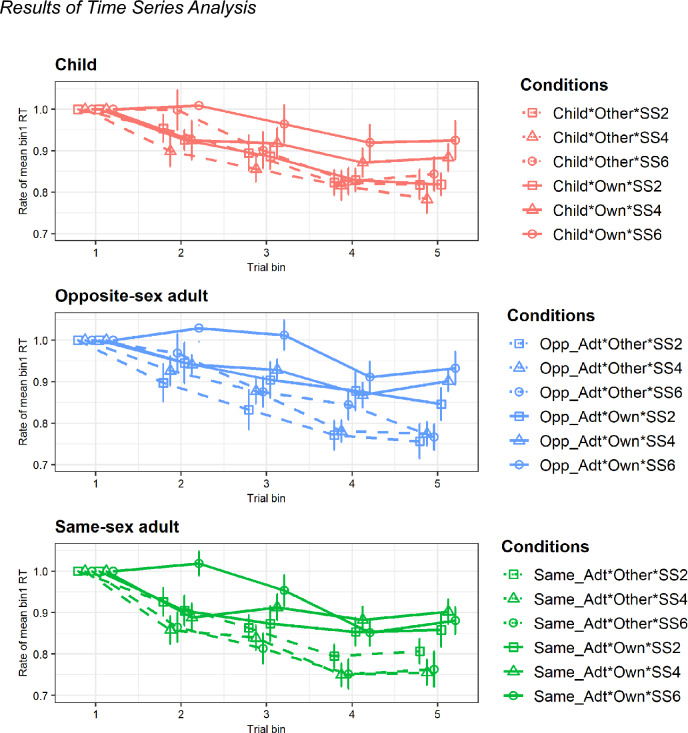
Results of time series analysis. *Note.* Neither the main nor the interaction effects of the sex of parent were significant. Therefore, the pooled results of mother/father conditions are shown. Error bars denote the standard error of the mean (SEM). Opp_Adt, Opposite-sex adult; Same_Adt, Same-sex adult; SS, Set size.

Table 3 presents an overview of the ANOVA results for the ratios of the bin1 means. A ratio of 1 indicates that there is no change between the mean RT of bin1 and that of the bin being compared, indicating no learning effect. A ratio smaller than 1 indicates that the mean RT of the compared bin is shorter than that of bin1, indicating a learning effect. We hypothesized that if participants overlearned their own and their family members’ faces, the ratios for their and family members’ faces would be larger than those for other-related faces over time (Fig. 4). For clarity, only significant results are reported here. The analysis revealed the main effects of ownership, *F*(1, 34) = 10.92, *p* < 0.005, *η*_*p*_^2^ = 0.24, set size, *F*(1.70, 57.90) = 3.86, *p* < 0.05, *η*_*p*_^2^ = 0.10, and trial bin, *F*(3.02, 102.53) = 90.53, *p* < 0.001, *η*_*p*_^2^ = 0.73. The trial bin-related two-way interactions were significant with ownership, *F*(2.71, 92.07) = 8.03, *p* < 0.001, *η*_*p*_^2^ = 0.19, and with set size, *F*(8, 272) = 2.75, *p* < 0.05, *η*_*p*_^2^ = 0.08. Post-hoc analyses with Bonferroni correction indicated that the learning effect from bin3 to bin5 was more prominent in the other conditions than in the own conditions (trial bin3: *p* < 0.01, Cohen’s *d* = 0.29; trial bin4: *p* < 0.005, Cohen’s *d* = 0.38; trial bin5: *p* < 0.005, Cohen’s *d* = 0.45, Fig. 4). Moreover, in the own condition, differences were significant except those between bin2 and bin3 and between bin4 and bin5 (*p-*values ranged from < 0.05 to < 0.001, Cohen’s *d* ranged from 0.20 to 0.86), and in the other condition, differences were significant except those between bin2 and bin3 (*p-*values ranged from < 0.005 to < 0.001, Cohen’s *d* ranged from 0.26 to 1.39). These results indicate that the tendency to shorten the RT was observed in both the own and other conditions, although this tendency was weaker in the own condition than in the other condition.

**Table 3:** Analysis of variance (ANOVA) of the rate of mean response time (RT) for Bin 1.

Source	Rate of mean RT for trial bin 1			
	*F* value	*df*	*p* value	Partial eta-square
Sex of parent (SP)	0.92	1, 34	0.34	0.03
Target type (TT)	1.33	2, 68	0.27	0.04
TT × SP	2.87	2, 68	0.06	0.08
Ownership (OS)	**10.92**	**1, 34**	**< 0.005**	**0.24**
OS × SP	0.001	1, 34	0.97	0.00
Set size (SS)	**3.86**	**1.70, 57.90**	**< 0.05**	**0.10**
SS × SP	0.43	1.70, 57.90	0.62	0.01
Trial bin (TB)	**90.53**	**3.02, 102.53**	**< 0.001**	**0.73**
TB × SP	0.61	3.02, 102.53	0.61	0.02
TT × OS	0.95	2, 68	0.39	0.03
TT × OS × SP	0.51	2, 68	0.60	0.02
TT × SS	0.78	4, 136	0.53	0.02
TT × SS × SP	0.34	4, 136	0.83	0.01
OS × SS	2.85	2, 68	0.07	0.08
OS × SS × SP	0.50	2, 68	0.61	0.02
TT × OS × SS	0.45	4, 136	0.74	0.01
TT × OS × SS × SP	0.98	4, 136	0.41	0.03
TT × TB	0.93	5.50, 186.98	0.47	0.03
TT × TB × SP	1.60	5.50, 186.98	0.16	0.05
OS × TB	**8.03**	**2.71, 92.07**	**< 0.001**	**0.19**
OS × TB × SP	1.26	2.71, 92.07	0.29	0.04
TT × OS × TB	0.47	8, 272	0.88	0.01
TT × OS × TB × SP	1.83	8, 272	0.07	0.05
SS × TB	**2.75**	**8, 272**	**< 0.05**	**0.08**
SS × TB × SP	1.21	8, 272	0.30	0.03
TT × SS × TB	0.54	9.09, 309.19	0.85	0.02
TT × SS × TB × SP	0.48	9.09, 309.19	0.89	0.01
OS × SS × TB	1.50	8, 272	0.16	0.04
OS × SS × TB × SP	0.22	8, 272	0.99	0.01
TT × OS × SS × TB	0.80	16, 544	0.68	0.02
TT × OS × SS × TB × SP	1.24	16, 544	0.23	0.04

Significant values are in bold.

### Correlational analyses between questionnaires and set size 1 RT

The results (Table 4) showed a significant positive correlation between the score of avoidance feelings toward infants and the set size 1 RT in the own-child condition for fathers (*r* = 0.72, *p* < 0.001, corrected by the Bonferroni method for multiple comparisons). This indicates that the stronger the avoidant feeling, the longer the search time for the child only for fathers. Correlations with the other scores were not statistically significant.

**Table 4:** Correlation matrix between questionnaires and set size 1 response time.

		1		2		3		4		5	
		Mother	Father	Mother	Father	Mother	Father	Mother	Father	Mother	Father
1	Set size 1 RT	–	–								
2	Care time on weekdays	0.18	− 0.07	–	–						
3	Care time on weekends	− 0.15	− 0.23	0.04	0.45	–	–				
4	Working hours	− 0.05	0.23	− 0.88	− 0.62	0.26	− 0.22	–	–		
5	Working days	− 0.01	− 0.05	− 0.90	− 0.16	0.17	0.32	0.93	0.12	–	–
6	Child care involvement	− 0.01	− 0.09	0.61	0.66	− 0.14	0.77	− 0.52	− 0.40	− 0.64	0.38
7	Parental affirmation	0.22	− 0.05	− 0.09	0	0.13	0.20	0.21	0.07	0.25	0.37
8	Parental denial	− 0.27	− 0.09	− 0.09	− 0.17	0.15	− 0.30	0.29	0.05	0.14	− 0.07
9	Approach toward child	− 0.19	0.32	0.31	0.35	− 0.03	− 0.18	− 0.21	− 0.16	− 0.32	− 0.34
10	Avoidance from child	0.16	0.72*	− 0.29	0.15	0.16	− 0.24	0.37	− 0.01	0.26	− 0.30
		Planned exploratory analysis		Supplementary exploratory analysis							

		6		7		8		9		10	
		Mother	Father	Mother	Father	Mother	Father	Mother	Father	Mother	Father
1	Set size 1 RT										
2	Care time on weekdays										
3	Care time on weekends										
4	Working hours										
5	Working days										
6	Child care involvement	–	–								
7	Parental affirmation	− 0.39	0.35	–	–						
8	Parental denial	0.20	− 0.38	− 0.24	− 0.10	–	–				
9	Approach toward child	0.46	− 0.02	− 0.24	0.25	0.33	0.19	–	–		
10	Avoidance from child	− 0.13	− 0.07	− 0.08	− 0.08	0.48	0.08	0.13	0.42	–	–
		Supplementary exploratory analysis									

*Indicates significant correlation (*p* < 0.05). Significant values are in bold.

## Discussion

This study investigated various aspects of finding one’s own child using a visual face search task. The RT analysis showed that as we find our own face more quickly than that of a stranger^7^, parents found their child’s face more quickly than other children’s faces. Moreover, the RTs for own and spouse’s faces were shorter than those for the faces of others of the same and opposite sexes. Accuracy analysis indicated a significant result: Parents found their child’s, spouse’s, and own faces slightly more accurately than those of non-family members. There were no significant differences in RTs or accuracy between the searches for their child’s, own and spouse’s faces. These findings indicate that own child’s face is perceptually special for parents, but those of their own and spouse are similarly special. Response facilitation and improvement of accuracy are observed not only for one’s own face but also for their child’s and spouse’s faces. A few previous studies with family members’ faces have reported that monozygotic twins can identify each other’s faces more efficiently than a friend’s face in a face identification task^27^. Meanwhile, face identification time did not differ among own, dizygotic twins, and stranger faces^28^. Thus, previous research using family faces has been limited to studies involving exceptional samples of twins. Therefore, the present study is the first to expand upon this research by including children’s faces and examining how parents process their children’s faces.

We performed time-course analyses to examine the possibility that quick search for family member faces was due to robust face representations owing to overlearning^7^. The time-course analysis with RT scaled as proportions of bin1 mean RT indicated that, in general, the other condition showed stronger learning effects than the own condition, and the learning effect between trial bins was less pronounced in the own condition than in the other condition. In other words, learning about family members’ faces progresses considerably more than that about others’ faces in daily life and may have reached a plateau. Consequently, the magnitude of decrease in RT across trials lessened. The participants may not reach the same level of learning as for the family members’ faces. Taken together, (over)learning of specific faces is critical for accelerating the search for one’s own family members’ faces.

RTs increased linearly with increasing set size in all conditions. Previous studies on visual search tasks have attributed the set-size effect to limited covert attentional processes^10^. Our results show that searching for one’s own and family members’ faces is an attention-demanded process, but not a pre-attentive process, such as a target face pop-out, regardless of the number of distractor faces. In line with these findings, Tong and Nakayama^7^ reported that RT for self-face search increased with the number of distractors. Moreover, Treisman and Gelade^8^ speculated that, “searching for a face, even as familiar as one’s own child, in a school photograph, can be a painstakingly serial process.” Thus, regardless of how special a person’s face, such as that of oneself, spouse, or child, it cannot be sought without attention.

Similarly, previous studies using infants' faces (but not their own children's faces) have reported visual processing specific to infants' faces, “attentional capture,” in which infant faces capture adults' attention (for a review, Lucion et al.^4^). For example, in the dot-probe detection task, participants can detect the dot probe faster when it appears near an infant's face compared to an adult's face or an animal’s face (dot-probe task, Brosch et al.^29^). In a free-viewing task, nulliparous adults have a longer fixation time for an infant's face than for an adult's face^30^. Future studies may report on the attentional capture of the faces of family members compared to the faces of unfamiliar strangers (see Devue et al.^31^ for attentional capture of own face). In an attentional capture task, bottom-up (stimulus-driven) attention is captured, whereas in a visual search task, top-down attention is directed toward a target. Bottom-up and top-down attention are independent^32,33^. Therefore, the issue of attentional capture by family faces will lead to a comprehensive understanding of how people process information related to their families in an environment overflowing with a vast amount of information.

Fathers and mothers showed different RT patterns between the child, same-sex adult, and opposite-sex adult conditions. Fathers found same-sex adults faster than they found opposite-sex adults and children; however, mothers did not. These results indicate that own-sex and own-age biases^21,34,35^ may be stronger for fathers. Additionally, mothers did not have own-age or own-sex bias. Although it is unclear why the paternal-maternal asymmetry arises in own-age and own-sex biases, the replicability of this asymmetry and its functional significance requires further detailed investigation.

Exploratory correlation analyses revealed that only fathers showed a correlation between RT for their child’s face and avoidance emotion score (*r* = 0.72); the stronger the avoidant feeling toward the child, the longer the RT for their child. These findings indicate that avoidance emotion toward children may be associated with a delay in finding their own child’s face. These results may be reflected in the difference in search time between the child and same-sex adult conditions, only in fathers. These results suggest that fathers’ avoidance emotion may hide in their exploratory behaviors toward their children. Such tendencies were not apparent in the mothers.

Although this study focuses on the differences in face information processing within families, such as fathers, mothers, and children, this study has a limitation that it does not allow us to know the difference between familiarity with family members and with people we know well, because there is no familiar control face condition. A familiar control face condition includes a familiar same-sex adult condition, a familiar opposite-sex adult condition, and a familiar child condition. Adding these control conditions can considerably increase the burden on participants. Furthermore, previous studies have mainly used face discrimination and identification tasks and have reported inconsistent results regarding familiarity with one’s own face and other familiar faces (friends’ or famous people’s faces). That is, the advantages of one’s own face over familiar faces are not always obvious. Previous reviews have suggested that cultural background should be considered^9,33^. The familiarity effects for own faces may be more pronounced in Western countries than in Eastern countries, and differences between own faces and familiar control faces are more evident among participants from Western countries than those from Eastern countries. Although cultural differences in the processing of one’s own or familiar faces are beyond the scope of this study, future research in this area is warranted.

In addition, we could have conducted a more rigorous controlled experiment by testing a completely unrelated control group with the same stimuli as in the present study. Although there has never been a completely unrelated control group in visual-search studies related to the self-face^7,36–38^, if ownership has no effect in the completely unrelated group but only on the father and mother, this will provide stronger evidence that the present results are based on familiarity rather than differences in physical characteristics. Despite the difficulty of conducting a controlled experiment in the present study because of the lack of experimental resources and the possibility that the results obtained may be complex and difficult to interpret, sufficient room for further investigation may remain.

The present study provides new insights into parents’ face-seeking behavior for their children. The search for the face of their own child was faster than that for other children's faces. The present findings support those of neuroimaging studies showing that parents, especially mothers, show specific brain activity for their children's faces (for a review, Rigo et al.^5^). However, similar phenomena were observed for their own and their spouses’ faces. In other words, we could not confirm a phenomenon that occurred only in the search for the faces of their children. Quick search for their child’s face may be based on overlearning of the faces of family members rather than caregivers’ attuned responses. However, the possibility of behavioral responses specific to their children’s faces could not be eliminated. Although the combination of own-age and own-sex biases may make interpreting the results difficult, it is necessary to conduct experiments such as searching for and finding a child among other family members. Future studies may clarify the special position of children among all family members and the specific treatment of children by parents.

## Data Availability

All raw data are available from the Open Science Foundation (OSF) repository at https://osf.io/5bzpg/.
